# Critical Analysis of the Evaluation Methods of Extended Reality (XR) Experiences for Construction Safety

**DOI:** 10.3390/ijerph192215272

**Published:** 2022-11-18

**Authors:** Daniel Salinas, Felipe Muñoz-La Rivera, Javier Mora-Serrano

**Affiliations:** 1 School of Civil Engineering, Pontificia Universidad Católica de Valparaíso, Valparaíso 2340000, Chile; 2 International Centre for Numerical Methods in Engineering (CIMNE), C/Gran Capitán S/N UPC Campus Nord, Edifici C1, 08034 Barcelona, Spain; 3 School of Civil Engineering, Universitat Politècnica de Catalunya, Carrer de Jordi Girona, 1, 08034 Barcelona, Spain

**Keywords:** construction safety, prevention of occupational hazards, virtual reality, augmented reality, mixed reality, extended reality

## Abstract

The construction industry has high accident rates. The sector is exploring various tools to improve safety management, training, and awareness to achieve zero accidents. This work focuses on extended reality (XR), which encompasses virtual reality (VR), augmented reality (AR), and mixed reality (MR) technologies. Several authors have developed training experiences for construction safety in XR environments with positive conclusions about their effectiveness. However, there is no standardization regarding the evaluation methods used in the sector, and many experiences do not use any method. This lack is critical, as whenever the aim is to evaluate the degree of awareness of security issues, the implementation of evaluation systems is indispensable to make known the methods used in the literature to evaluate the effectiveness of the experiences and represent support for future research. This research identifies developments in XR experiences and analyzes the validation methods through a systematic review using the PRISMA methodology. It identifies two evaluation methods, objective and subjective, which are each broken down into four categories. The results show the types of evaluation, safety-related purposes, and safety application objectives used by the database classification.

## 1. Introduction

### 1.1. Construction Safety

The construction industry is characterized by safety issues [1] due to weather issues, the use of heavy machinery and transportation of materials, the interaction of multiple agents in a project, and their integration in the different stages, which create situations where the safety of the construction site becomes more difficult to control. The construction industry in the USA accounted for 21.6% of all fatal injuries in 2019 [2]. The equipment orientation aspects in companies are poor due to a lack of coordination between workers, supervisors, and management, including cooperation and communication between management and workers about the work, site, health, and safety. In addition, inadequate use of personal protective equipment (PPE) and handouts and low interest in reading safety manuals contribute to accidents. Managers should establish safety policies for their company and train employees to achieve the goal of zero accidents. All parties in the construction project must contribute their fair share of effort to make construction sites healthy and safe [3].

Researchers are testing several tools to improve safety in construction, integrating methodologies and technologies to improve management, processes, and training of workers at different stages of construction projects [4]. In particular, prevention through design (PtD) integrates safety aspects from the early design stage of projects [5]. The lean construction methodology encourages process identification, reduction, and optimization [6]. The building information modeling (BIM) methodology seeks to integrate safety processes and elements into multi-dimensional models of projects [7].

Information technologies improve the configuration of workspaces at the site and the safety procedures of a construction project [8]. Worksite sensing technologies measure movement, temperatures, body sensations, etc. They allow the monitoring of worker behavior and their interactions with heavy machinery and equipment to control work dynamics, study behaviors, and avoid accidents [9]. Interconnected work with BIM models and processes enables better safety planning and control based on digital multi-parameter models. The connection of BIM environments with job site sensors and simulations of worker and machinery behavior represents an advance in risk prevention tools for the design, management, and training of workers. However, BIM environments lack immersive tools that allow for deeper analysis of safety behaviors prior to the execution of the works for design reviews, safety planning, and worker training, among other requirements. The use and connection of these sensors with simulations in BIM environments represent an advance in risk prevention tools for the design, management, and training of workers. One tool that has grown significantly in research in recent years is the extended reality (XR) for construction safety. This tool encompasses virtual reality (VR), augmented reality (AR), and mixed reality (MR) technologies [10]. These tools are becoming increasingly important in various industries, particularly in the construction sector, as they allow the immersion and recreation of realistic environments, facilitating the review of projects, training, the inspection of protection elements, and providing useful functionalities for safety management [11]. These, in turn, can use and incorporate BIM models and data (tools used in the construction sector), facilitating their incorporation into the construction sector.

### 1.2. Extended Reality (XR) for Construction Safety

Virtual reality (VR) enables the creation of an interactive environment for cognitive learning where users can examine virtual but realistic site environments, identify hazards, and perceive the direct consequences of their actions without exposing themselves to the risks of reality [12,13]. On the other hand, augmented reality (AR) technology overlays the view of a real-world physical environment with objects in a virtual world [14], enabling construction project scheduling, progress tracking, worker training, safety, time, cost, quality, and defect management [15]. Mixed reality (MR) is the hybrid technology between digital reality and virtuality, combining real and virtual elements to form a continuous cycle of interaction between the real world and digital construction information [16].

These technologies enable immersive project review, virtual and safe project walk-throughs, incorporation of safety elements (either personal protection elements or general safety elements for the construction site), and the management of collective and individual protection elements on the job site, among other uses [17]. Some of the most common XR experiences deal with safety issues, such as working at heights [18,19,20], the influence of noise on workers’ decisions [21,22,23], monitoring through the use of augmented reality and sensors to track the interaction between heavy machinery and workers [9,24,25], and mainly in training on the use of tools, machinery, materials, among others [26,27,28]. For training activities, virtual reality (VR) educational narrative games provide rich, high-fidelity environments that deliver a fully immersive and interactive storytelling experience for teaching [29]. These virtual environments, also called ‘serious games’ [30], combine with XR experiences for realistic and immersive experiences. Multi-sensory experiences require devices, such as virtual reality goggles and sensors [31], control handles [32], and many other tools that are programed to link with the developed XR experience.

### 1.3. Knowledge Gap and Research Objectives

This work reviews the authors who have worked in different areas of the construction industry using XR experiences, particularly those addressing various aspects and functionalities affecting safety. None of these studies analyzed the techniques used to validate the methods effectively. This analysis is essential to measure the level of success of an XR experience and to identify the shortcomings of the experience to improve it and reach the expected final goal. This research is important because it identifies and defines the methods and types of evaluation in the literature over the past 12 years. This could serve as a guide and support for future research related to XR experiences. Having clear methods and types of evaluation facilitates the planning, development, and validation of XR experiences. In cases where an XR experience has been developed but lacks evaluation, it is impossible to establish whether the experience meets its objectives of usability, applicability, and understanding by users, among other variables. Therefore, the aim is to diagnose which evaluation methods have measured the effectiveness of extended reality experiences.

Given this knowledge gap, this research sets the following objectives:

- Identify the XR evaluation methods and understand the categories, typologies, and criteria to classify and understand the evaluation methods. Additionally, understand how these methods have addressed the aspects of security and extended reality experiences.
- Understand the differentiation and the methods the experiences use according to the purposes of construction safety.
- Identify how these methods vary according to security-related purposes and security application objectives.

Next, this research presents the methods for evaluating the effectiveness of XR experiences to judge the different types of experiences optimally and to define a standardization of techniques for the evaluation of construction safety through XR experiences in subsequent research.

## 2. Methodology

This research identifies and analyzes the methods used to evaluate the effectiveness of extended reality (XR) experiences for construction safety, according to scientific publications describing these experiences. This paper uses a mixed review method, i.e., quantitative and qualitative review, to achieve its objectives, comprising bibliometric and systematic approaches, combining the advantages of both to eliminate subjective interpretations and biased conclusions and understand the domain of knowledge and research trends. It examines a set of articles selected under criteria that link them to the objectives of this research. It performs a statistical analysis of the trends found within the articles reviewed to achieve a more complete analysis. The research methodology contains two stages: (1) the extraction and evaluation of bibliometric data and (2) the identification and analysis of evaluation criteria and classification of XR experiences.

### 2.1. Bibliometric Data Extraction and Evaluation

**Figure 1:**
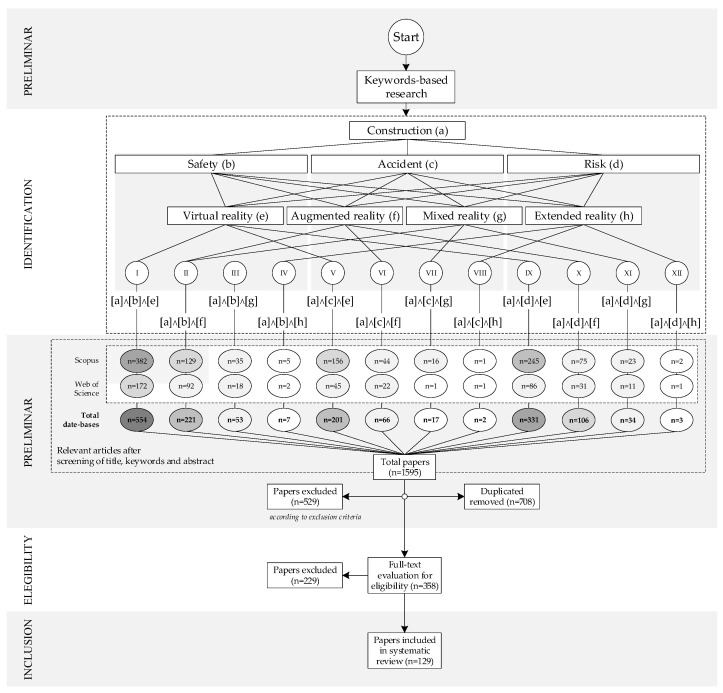
PRISMA workflow for systematic review.

This work used the Preferred Reporting Items for Systematic Reviews and Meta-Analyses (PRISMA) [33] methodology to search and select the articles. Figure 1 shows the PRISMA workflow for the systematic review. This methodology indicates the keywords, database libraries, and inclusion and exclusion criteria to filter the retrieved studies.

#### 2.1.1. Database and Keyword Selection

The first phase of the PRISMA methodology used the Scopus and Web of Science databases due to their wide variety of scientific articles and their international prestige in similar research. The following keywords were used for the preliminary search of the articles to be used: [a] Construction; [b] Safety; [c] Accident; [d] Risk; [e] Virtual Reality; [f] Augmented Reality; [g] Mixed Reality; and [h] Extended Reality. These words were discussed and agreed upon by the authors of the research, and they were considered more representative of the topic and objectives addressed within the research and sufficient to fulfill and summarize the defined research requirements. For these keywords, 12 search combinations were performed: (I) [a]∧[b]∧[e]; (II) [a]∧[b]∧[f]; (III) [a]∧[b]∧[g]; (IV) [a]∧[b]∧[h]; (V) [a]∧[c]∧[e]; (VI) [a]∧[c]∧[f]; (VII) [a]∧[c]∧[g]; (VIII) [a]∧[c]∧[h]; (IX) [a]∧[d]∧[e]; (X) [a]∧[d]∧[f]; (XI) [a]∧[d]∧[g]; (XII) [a]∧[d]∧[h].

#### 2.1.2. Inclusion and Exclusion Criteria

The next phase defines the inclusion and exclusion criteria, which filter the articles found in the previous one. Inclusion criteria include: (1) studies that focus on the use of virtual, augmented, mixed, or extended reality for construction safety; (2) studies that show specific applications or developments with different objectives; (3) studies published in peer-reviewed journals and conference proceedings; and (4) articles between 2010 and 2022. Exclusion criteria are: (1) theoretical studies or literature reviews that do not show specific developments in VR/AR/MR/XR for construction safety; (2) studies published in languages other than English; and (3) studies with no full text available.

#### 2.1.3. Screening and Evaluation of Studies

Taking the above into account, up to March 2022, 1595 articles were found in the Scopus (1113) and Web of Science (482) databases. Figure 1 shows all the papers obtained for each search combination and database. The second ‘PRELIMINARY’ phase eliminated 708 duplicate articles. Using the PRISMA methodology, a review of the titles, keywords, and abstracts of the remaining articles excluded 529 articles for not meeting the criteria established above. Then, the ‘ELIGIBILITY’ stage of Figure 1 examined the remaining 358 complete papers, and then, 229 that were considered irrelevant to the research objectives were excluded. Thus, a total of 129 articles were selected for analysis.

### 2.2. Identification and Analysis of the Evaluation Criteria and Classification of XR Experiences

The literature review carried out a qualitative analysis of the selected articles. This analysis aimed to determine how researchers evaluated the experiences developed in XR. The secondary objective was to understand the purpose that motivated the authors to conduct their research, the methods of applying the XR technologies, and how to evaluate the effectiveness of the developed experiences. Based on the quantitative results of Section 2.1, this research studied the works related to its objectives. The criteria for classifying the articles are defined, followed by a statistical analysis and conclusions that allow an understanding of the types of evaluation used in the various experiences. The main methods and types of effectiveness evaluation, purposes, and application objectives related to safety are studied. Furthermore, this work analyzes trends to study the aspects of safety in construction and XR technologies. It performs the following analyses.

#### 2.2.1. Characterization of the Reviewed Studies According to Extended Reality (XR)

First, the research identifies the extended reality technologies used in the experiences of the database, i.e., whether it uses virtual, augmented, or mixed reality, and it quantifies the use of these technologies by year of publication. In addition, an analysis verified whether or not the research evaluated the experience to identify the studies that will be used to carry out the statistical analysis and classification of the database by defining the criteria.

#### 2.2.2. Criteria for Literature Classification

This work created criteria to classify the articles depending on how to evaluate the effectiveness of the developed experiences. These criteria are: (1) methods and types of effectiveness evaluation; (2) safety-related purpose; (3) safety application objective; and (4) evaluation performed within or outside the XR experiences.

## 3. Results and Analysis

The results from the bibliometric study, literature review, and statistical analysis performed with the selected databases appear below. In addition, the main characteristics and trends of the articles reviewed appear.

### 3.1. Overall Characteristics and Results Found in the Literature

This section presents the main results corresponding to the quantitative characteristics of the literature concerning the year of publication, country of origin, distribution of articles by the journal in which they were published, and technologies used by year of publication.

#### 3.1.1. Annual Quantitative Distribution of Literature

**Figure 2:**
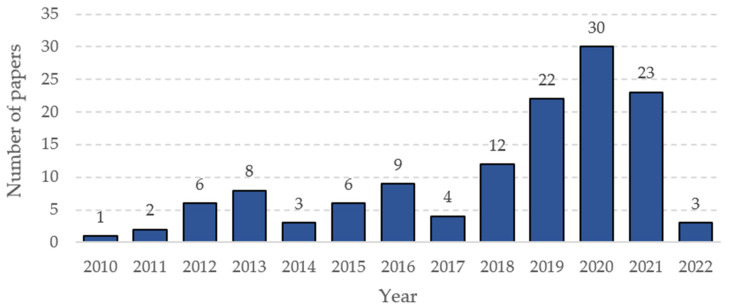
Number of articles by year of publication.

Following the research methodology and established criteria, 129 articles were selected for the literature review. Figure 2 shows the annual distribution of the 129 articles analyzed between 2010 and 2022. The database search ended in June 2022, so the number of papers found for the current year is low. The distribution tends to rise, increasing substantially between 2019 and 2021, so this amount will likely be equal to or higher for 2022 when the database is incomplete. In 2010 and 2011, the number of articles published was minimal. However, from 2012 onwards, there was an increase in the number of papers related to XR technologies. This trend is evidence of the increase in research on these tools.

#### 3.1.2. Quantitative Analysis of Main Countries of Origin

**Figure 3:**
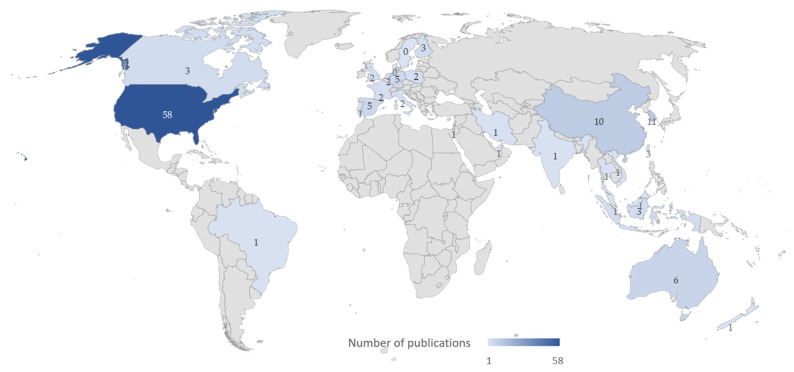
Distribution of published articles by country.

According to the results and the database, Figure 3 shows a map of the countries that conducted the identified research works. The countries with the highest number of publications appear in dark tones, with lighter colors decreasing as the number of publications decreases. The countries with the highest number of scientific articles published are the United States (45%), South Korea (8.5%), and China (7.8%). The number of publications from the United States is considerably higher than from other countries.

#### 3.1.3. Quantitative Analysis of the Main Journals and Conferences

The 129 articles selected for this research came from 36 journals and 35 conference proceedings. Table 1 shows the primary journals and conference proceedings identified and the number of articles published, representing 62% of the total number of publications studied. The research topics of the journals and conference proceedings are related to construction, engineering, computer science, technological applications, health and safety, and sustainability, among others. Within these general or particular fields, academic sources focus on these topics in the architecture, engineering, and construction industries.

#### 3.1.4. Distribution of Technologies Used by Publication Year

**Figure 4:**
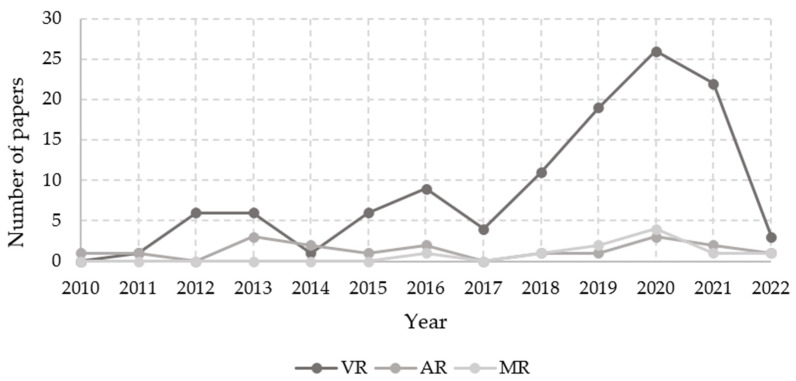
Number of articles in relation to the technology used by year of publication.

Of the total of 129 articles, Figure 4 shows the number of studies each year related to the technologies of interest, either VR, AR, or MR. VR was the most used over the years compared to the other technologies, and it presented an increasing trend, which increased considerably from 2018 onwards. The AR and MR technologies were used less frequently in the studies, having an almost linear trend over the years. Some articles addressed more than one technology. The figure shows that 88.4% of the studies used virtual reality (114 articles), 14% used augmented reality (18 articles), and only 7.8% used mixed reality (10 articles).

### 3.2. Methods to Measure the Effectiveness of XR Experiences for Construction Safety

Next, this research analyzes the criteria related to the evaluation of the experiences carried out in each article of the database. At this stage, the number of articles analyzed is low, since it only considers those articles that evaluate the experience. In addition, it points out why some documents do not evaluate such experiences. The classification of the evaluations concerning the other criteria considered is also mentioned, including the evaluation methods, types of evaluations, purpose related to the safety of the XR experiences, and the safety application objectives of such experiences. A statistical analysis of the criteria shows the trends in the literature.

#### 3.2.1. Analysis and Screening of Selected Studies

**Figure 5:**
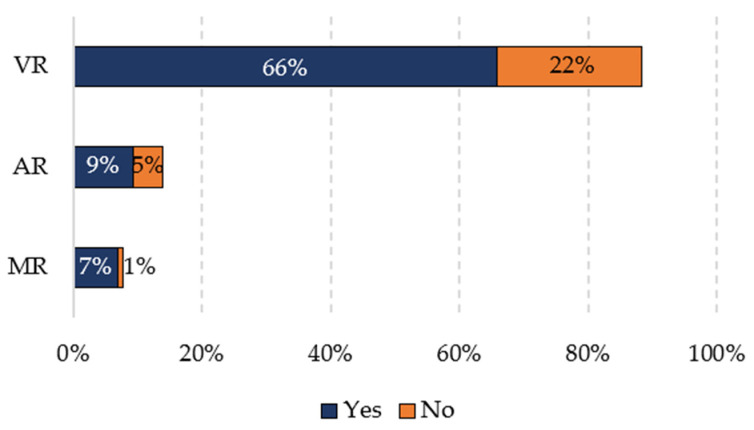
Number of studies that use or do not use some method to measure the effectiveness of XR experiences according to the technologies used.

Of the total of 129 articles, 97 evaluate the XR experience for safety, i.e., approximately 75.2% measure the effectiveness of the experience developed. Figure 5 shows the number of studies that use some method to measure the effectiveness of XR experiences according to the technologies used. It is possible to see that the trend of the general results remains the same: VR technology stands out above the others. It is evident that for virtual reality, a total of 65.9% of articles perform an evaluation (85 articles) and 22.5% do not (29 articles); for augmented reality, 9.3% perform an evaluation (12 articles) and 4.7% do not (6 articles); for mixed reality, 7% perform an evaluation (9 articles) and 0.8% do not (1 article).

The primary reason identified for the 32 studies that do not consider methods to measure the effectiveness of their experiences is that the focus has been on the development of the XR experience, both conceptually and technologically. For example, they describe the algorithm or the simulation of the experience without evaluating its effectiveness. This also denotes the novelty and complexity of the development of these experiences, especially for non-expert or non-professional users, whose work focus is the discipline of technology application and not in the XR technology area as such.

Within the literature that does not perform evaluation, most of the XR developments for safety focus on the design, development, and simulation of the experience. These include topics such as: training in the use and operation of cranes [34,35], postural training [36], paving operations [37], among others [38,39,40,41,42,43]; collision detection [44,45]; prevention of occupational hazards [46,47,48,49,50]; and the general topics of construction safety [51,52,53,54,55,56]. Some papers test the operation of the technology in addition to focusing on the development of the experience. However, they do not evaluate the effectiveness or provide details [34,36,48,50]. On the other hand, some articles mention the need to perform evaluations within their future research [37,57,58,59,60,61,62,63,64]. Measuring the effectiveness of XR experiences is of utmost importance to estimate the knowledge acquired by users in training and to measure the usability of the experiences performed.

Therefore, the articles in the database that do not perform an evaluation are incomplete for the purpose of this research. The values, percentages, and data presented below include only those articles that are evaluated, thus reducing their number from 129 to 97.

#### 3.2.2. Methods Identified to Measure the Effectiveness of XR Experiences

Researchers have identified various methods to measure the effectiveness of XR experiences. These methods verify the functionality and effectiveness of the experiences, i.e., they test the capacity to produce the effect of the experiences desired by the authors. For example, the authors established certain achievement objectives (knowledge, skill in performing a task), and the users achieved satisfactory evaluations according to the parameters defined for each experience. Practical uses, such as safety facility selection, education requirements, work sequence adjustment, and safety management, can not only conceptually set goals but measure them against extended reality experiences [65]. User experience measurement is similar to usability measurement, where user behavior data within experiences can serve as metrics of XR effectiveness [66]. Other cases, for example, compare the performance or motivation of learners when using XR experiences versus a traditional instructional medium. This method measures students’ performance when taking a test to measure content mastery. Thus, they compare the scores of students belonging to the experimental group (receiving the XR technology intervention) and the control group (receiving a traditional instructional medium) to detect differences in learning [67].

**Figure 6:**
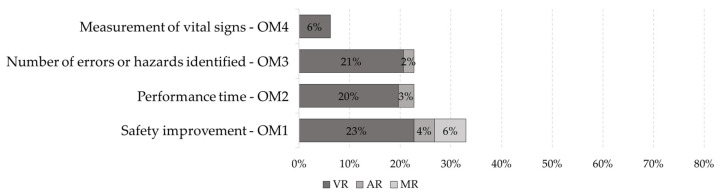
Types of evaluation belonging to the target objective method and number of corresponding XR tools.

**Figure 7:**
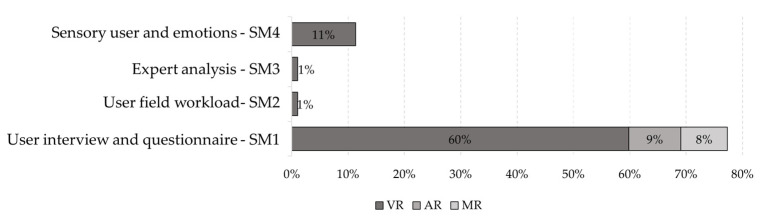
Types of evaluation belonging to the subjective method and number of corresponding XR tools.

Li et al. [65], based on a literature review of XR for the AECO industry, identify categories of evaluation methods for these applications. Based on these methods, this research takes the proposal of Li et al. [65] as the basis. We adapted and complemented it with the work of this research. The two main categories are objective methods and subjective methods. Objective methods are rigorously and concretely elaborated measurement or evaluation instruments. They use measures predisposed or defined by the authors, including established data, expected actions, or quantitative results, such as the number of errors or hazards identified. Subjective methods are measurements or evaluation instruments that vary between two or more participants when considering the same activity, behavior, or opinion. They include questionnaires, where the response varies depending on the performance or opinion of the participant. They depend on what is to be evaluated, including usability, effectiveness, applicability, emotions, or sensations. Figure 6 shows the findings for the subjective methods and Figure 7 shows the findings for the subjective methods.

Objective methods include four types:

(1) **OM1—Safety improvement.** This consists of evaluating the correct behavior under specific circumstances that improves and ensures safety in the processes or activities within the construction site, for example, training to educate about the ideal posture when performing tasks and thus avoid accidents or diseases. In this case, the evaluation compares the postures of the participants within the practices and the recommended postures stored in the system database to deliver additional learning material if they are incorrect [68]. Another example is to monitor the site environment to avoid collisions or warn of approaching hazards. A case is evaluated by collecting live field data, filtering for unsafe situations, processing, and finally transmitting safety information in real time to a virtual reality. This information is then compared by including sensors to avoid accidents within the simulations, thus decreasing the number of unsafe situations to which workers are exposed [9,69]. Another case incorporates the warnings of proximity to hazards, and the decrease in accidents occurs within various case studies [69]. This type of evaluation seeks to quantify the safety of the construction site before and after the proposed experience, i.e., to verify the improvement or increase in safety compared to the non-use of the experience. Thus, Hasanzadeh et al. [70] use three levels of protection in a roofing activity and evaluate how it affects the feeling of safety in the participants’ decision making, thus observing and comparing the level of safety in the different cases. This type of evaluation appeared in 27.8% of articles (27 articles). In addition, concerning the technologies, 22.7% used VR (22 articles), 4.1% used AR (4 articles), and 6.2% used MR (6 articles).
(2) **OM2—Performance time.** This consists of measuring the time taken by participants to complete a task [71,72] and the time intervals between activities or subtasks [73], among other cases of XR experiences. The time helps to obtain trends and draw conclusions based on the time obtained by each participant. The questions in this type of evaluation are what influenced that time and what consequences this brings for safety at the construction site. An example is when an activity uses eye tracking. Thus, it measures the time it takes the participant to fixate the gaze on an object and the time the gaze remains on that object [74,75]. This type of assessment occurred in 20.6% of articles (20 articles). Additionally, concerning technologies, 19.6% used VR (19 articles), 3.1% used AR (4 articles), and 0% used MR (0 articles).
(3) **OM3—Number of errors or hazards identified**. This measures the number of errors made by the participants in the XR experiences or the number of hazards identified in the simulations. In this way, it compares the numbers obtained by the participants, and a critical analysis leads them to these values. Some cases in the literature train participants in risk identification as if they were on the construction site. Shamsudin et al. [76] selected a group of trainees and asked them to identify the health and safety hazards. They evaluated these hazards based on their frequency and accuracy. This technique quantified the hazards and compared them with the hazard identification report performed in a real construction project. Lu et al. [22] used the number of errors made by the participant as a penalty by deducting time from the time limit, thus evaluating the participant’s performance. This type of evaluation occurred in 22.7% of articles (22 articles), and concerning technologies, 20.6% used VR (20 articles), 2.1% used AR (2 articles), and 0% used MR (0 articles).
(4) **OM4—Measurement of vital signs.** This monitors the vital signs of participants by heartbeat, respiration, or perspiration, among others. These cases use tools, such as electrocardiograms or electroencephalography, to measure the physical response of the participant under various stressful circumstances or activities. Xu et al. [77] used electromyography and a heart rate sensor to reveal instantaneous muscle strains caused by fear during VR training on fall prevention from height. Habibnezhad et al. [78] used a Fitbit Versa health smartwatch, which measured the heart rate while performing activities at heights in an immersive VR environment. Another example is the research by Huang et al. [32] and Jeon et al. [79] where they used an encephalography system, and Huang et al. recorded the heart rate and blood pressure while simulating six major injury scenarios within the construction site. Kim et al. [80] collected and analyzed biosignals from participants, such as electrodermal activity, pupil dilation, and saccadic eye movement. This type of assessment occurred in 6.2% of articles (six articles), particularly in VR. For AR and MR, no publications were identified.

On the other hand, subjective methods include four types:

(1) **SM1—User interview and questionnaire.** This method uses surveys, questions, and questionnaires to measure the knowledge, opinion, or thinking of the participants about the experience, usually using both open and closed questions, including multiple-choice alternatives. This method evaluates the usability, applicability, and effectiveness of the experiences considering the participants’ responses. In addition, it is the type of evaluation most commonly used in the database analyzed. Within the literature, articles were identified that use a means to evaluate experiences: interviews [19,24,27,68,70,73,81,82,83,84,85,86,87,88,89,90,91,92]; questionnaires [13,21,22,23,25,93,94,95,96,97,98,99,100,101,102,103,104,105,106,107]; interviews and questionnaires that use the Likert scale [108] to quantify the participants’ response, varying between five, six, and seven values [26,28,32,71,80,109,110,111,112,113,114,115,116,117,118,119,120,121]; multiple-choice or true–false questions [81,99,113,114,122,123]; surveys [30,83,123,124,125,126,127,128]; and open-ended questions [26,98,129]. This type of evaluation occurred in 69.1% of articles (67 articles). Concerning the technologies, 59.8% used VR (58 items), 9.3% used AR (9 items), and 8.2% used RM (8 items).
(2) **SM2—User field workload.** This focuses on measuring or understanding how workers’ perceived cognitive load influences their performance of activities within the construction site, such as fatigue, work pressure, or stress. These conditions can be assessed using body measurement tools, such as an eye tracker, electrocardiogram, pulse oximeter, or temperature. This type of assessment occurred in only one article in the database, where Han et al. [128] used two complementary methods to measure cognitive load. It included (1) a questionnaire for self-assessment of cognitive load. They used NASA-TLX [130] as a multi-dimensional measurement method due to its adequate level of sensitivity and complexity and its widely proven reliability in measuring the cognitive load of individuals. It also included (2) the performance of tasks performed, considered an objective tool for measuring cognitive load. It is characterized by observing how time pressure affects the accuracy rate when performing tasks. It showed that time pressure undermines task performance, creating uncertainty in the individual performances of participants. Another example of this type of assessment would be to evaluate the cognitive load of workers when exerting too much pressure by measuring the level of fatigue or stress using surveys or body measurement tools. This type of assessment occurred in 1% of articles (one article), particularly in VR. For AR and MR, no publications were identified.
(3) **SM3—Expert analysis.** This indicator verifies the functionality and effectiveness of the experience through the opinion and critical analysis of experts. Professionals or experts can measure it by participating directly in the experience, observing the characteristics and performance of users within it, or participating in the design and parameterization of XR experiences. For example, experts could help determine the factors to be developed by technical managers and validate the technology, experience, and user behavior. Within the database, one article showed where expert analysis facilitated experience evaluation. Raimbaud et al. [92] used AEC industry professionals to define the experience quality factors to evaluate. The study selected the following quality factors for evaluation: speed, information-gathering capability, ease of use, and spatial awareness. This type of evaluation occurred in 1% of items (one item), particularly in VR. For AR and MR, no publications were identified.
(4) **SM4—Sensory user and emotions.** This indicator focuses on identifying the emotions or sensations perceived by participants in dangerous situations or situations that could become unsafe or stressful for users. In some cases, the sensors and body trackers serve as measurement tools, such as sensors for gloves, arms, legs, and eyes, where the behavior or movements of the participants may vary during the experience. Habibnezhad et al. [17] used body trackers to measure participants’ gait or movement and assess the fall risk. Other articles in the database perform hazard recognition by eye tracking of participants [74,131]. Additionally, within the literature, studies measure how sensations affect the safety and productivity of construction workers [18,132]. Other studies assess fear to determine low-risk behavior on elevated surfaces [78,133]. Fear induction can also be measured using electromyography, which measures the degree of emotional arousal [77]. Gao et al. [134] measured workers’ personality traits, such as extroversion, agreeableness, conscientiousness, and neuroticism, to predict their safety behavior. This type of assessment appeared in 11.3% of items (11 items), all relating to VR.

#### 3.2.3. Safety-Related Purposes of XR Experiences

Frank Moore et al. [135] identified the primary purposes related to construction safety and the safety application objectives of the research. For the development of this research and based on the identified findings, their proposal was taken as the basis, adapting and complementing it with the work of this research. Concerning the purposes, it defines three categories:

- **Education and training.** This purpose is the primary one analyzed. In these cases, the XR experiences instruct and train students or workers in construction safety in different practices, such as the use of machinery, identification of hazards, or correct behavior in various situations, for example. Within the literature, several authors have used this purpose for their research. Among them, we can find training in postural balance [18,136,137], excavation processes [124,138], training oriented to improving safety within the construction site [17,27,71,104,105], training in hazard identification [76,139,140,141,142], and training in the use of personal protective equipment [82]. Other more specific examples are the article by Barkokebas et al. [143], which developed an experience for training in the assembly, disassembly, and maintenance of construction machines, and the article by Shi et al. [20], which investigated the social learning behavior of people in a hazardous construction situation. This safety-related purpose occurred in 86.6% of articles (84 articles). Concerning technologies, 77.3% used VR (75 articles), 7.2% used AR (7 articles), and 8.2% used MR (8 articles).
- **Monitoring of the site environment.** These cases use XR tools to monitor and manage safety on the construction site, for example, using sensors, which avoids collisions or accidents on the construction site. Monitoring the on-site environment can considerably improve site safety and facilitate communication and information transfer. The literature identifies cases in which sensors monitor the position [9] and body movements, such as head or ankle [100], while others develop warning systems to warn of the proximity of hazards or collisions on the site [69,102]. Some articles sought to monitor the environment and improve the transmission of data and information [9,24] and study the interaction between agents involved in an activity [25]. In addition, one article focused on monitoring the environment interacting with machinery [101]. This safety-related purpose occurred in 8.2% of articles (eight articles). Concerning technologies, 5.2% used VR (five articles), 3.1% used AR (three articles), and 1% used MR (one article).
- **Safety planning.** This type of experience allows effective safety planning to avoid risks before and during construction processes. The analyzed literature contained a variety of cases with this purpose. Alvanchi et al. [129] proposed a method to plan and manage the safety of construction projects during the commercialization process. Pooladvand et al. [144] investigated a crane simulator that integrates lifting survey data and a detailed crane route planning system. Other studies investigate immersive environments for protection system selection and guardrail design in construction projects [111,132]. Park et al. [84] proposed a safety management and visualization system that integrates building information modeling, location tracking, augmented reality, and gaming technology. This security-related purpose occurred in 5.2% of articles (five articles). Concerning technologies, 5.2% used VR (five articles), 2.1% used AR (two articles), and 0% used MR (zero articles).

**Figure 8:**
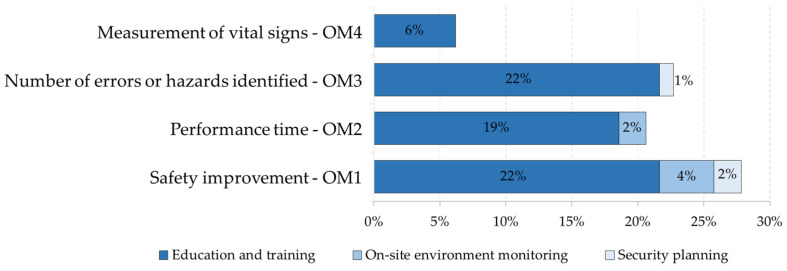
Number of items for each safety-related purpose by type of objective method assessment.

**Figure 9:**
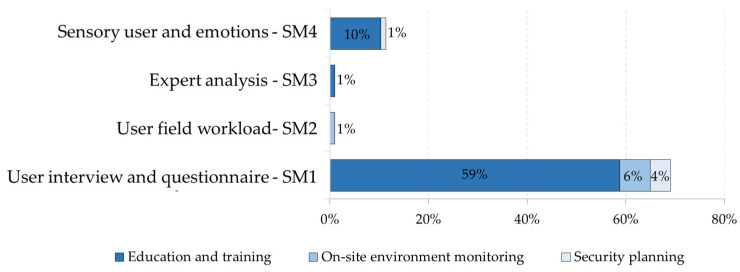
Number of items for each safety-related purpose by type of subjective method evaluation.

Figure 8 and Figure 9 show the relationship between the purposes and the different types of evaluation identified. The percentages are approximate to unity. The most used purpose in the literature and all types of evaluation is ‘Education and training’.

#### 3.2.4. Safety Application Objectives in XR Experiences

**Figure 10:**
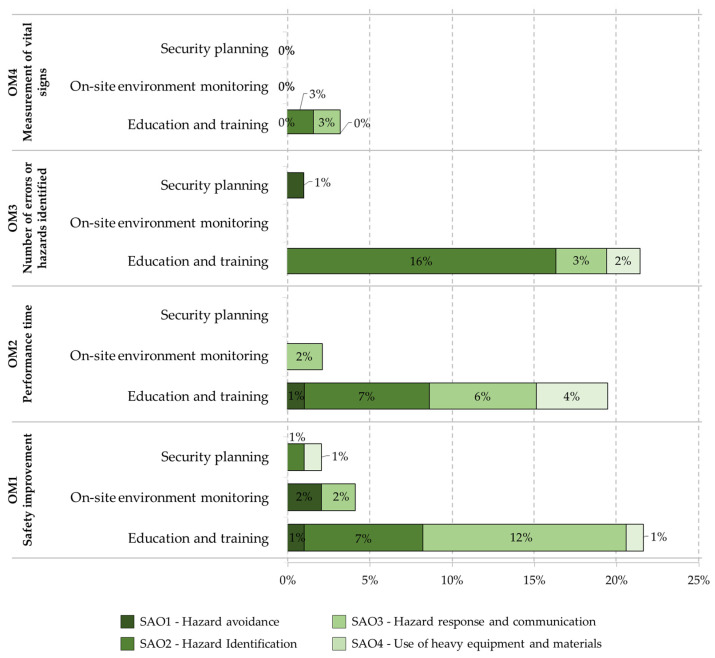
Number of items of each safety application objective for each purpose related to the types of evaluation of the target method.

**Figure 11:**
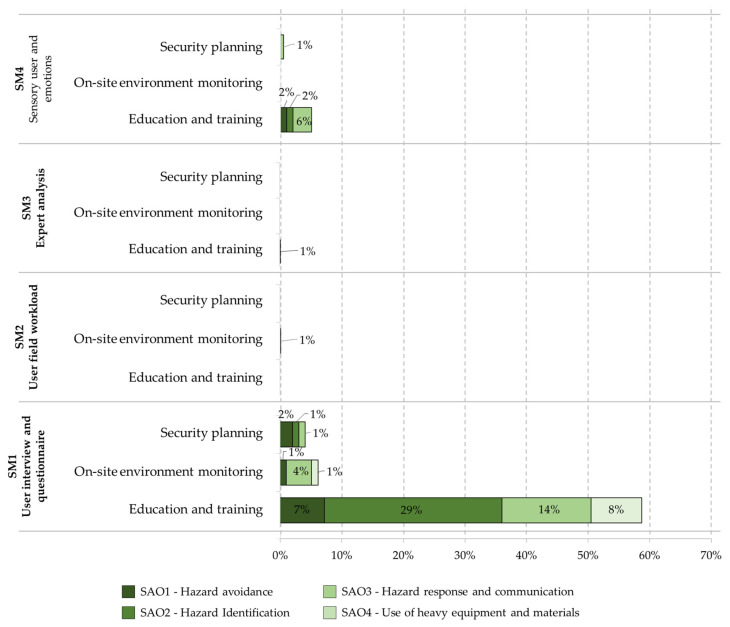
Number of items of each security application objective for each purpose related to the types of evaluation of the subjective method: User interview and questionnaire; User field workload; Expert analysis; and Sensory user and emotions.

As mentioned in Section 3.2.3, the safety application objectives use our adaptation of the Frank Moore et al. article [135]. The following objectives are defined and implemented within the literature related to safety within construction. Figure 10 show the number of items of each safety application objective for each purpose related to the types of evaluation of the target method, and Figure 11 show the number of items of each security application objective for each purpose related to the types of evaluation of the subjective method.

- **SAO1—Hazard avoidance.** As its name suggests, this safety application aims to prevent risk situations through training, environmental monitoring, or proper safety planning at the construction site. In this line, we identified the following cases in the literature, including the learning and evaluation of the risk of falls from heights to improve workers’ safety awareness [17,27]. Brioso et al. [111] used an XR experience to select a fall protection system. Simeonov et al. [18] studied the effect of workers’ balance by using plantar vibrations, thus improving safety at heights. Other studies focus on monitoring the environment, thus developing hazard proximity warning systems [9,69,102]. Alvanchi et al. [129] developed a method to address the safety problem of construction projects in the marketing process. Other cases that reflect this safety enforcement objective [71,82,104,105,121,124] focus on improving safety within the construction site. This safety application objective occurred in 14.4% of articles (14 articles). Concerning technologies, 13.4% used VR (13 articles), 4.1% used AR (4 articles), and 1% used MR (1 article).
- **SAO2—Hazard identification.** This safety application uses XR technology to facilitate hazard identification by creating immersive training environments or a risk recognition technology. Some of the experiences identified in the literature include research using eye tracking to study the cognitive process of participants when identifying hazards [75,145]. Perlman et al. [146] asked participants to identify hazards in a typical construction project, assess their level of risk, and estimate the probability and severity of potential accidents. Hilfert et al. [147] conducted a study where participants had to transport objects from one point to another and identify an excavator that was approaching them, using sound and sight to detect the hazard. Golovina et al. [148] developed an experience where previously unknown data are recorded and analyzed by identifying close calls and contact collisions that put participants at risk. Most research aims to generate experiences and simulations where participants must identify hazards [76,140,141,142,149]. This safety application objective occurred in 45.4% of articles (44 articles). Concerning the technologies, 41.2% used VR (40 articles), 6.2% used AR (6 articles), and 1% used RM (1 article).
- **SAO3—Hazard response and communication.** This type of safety application aims to measure or visualize the user’s response and behavior to hazardous situations and observe how the XR technology facilitates communication within the construction site. We have the following cases to make this safety application objective clearer: studying the response of participants to hazardous situations, such as working at heights [20,137], and studying postural balance by measuring the center of pressure and upper body sway motion under various inclined angles at heights [2]. Olorunfemi et al. [28] evaluated an MR technology to improve workplace safety hazard communication. Kim et al. [80] evaluated whether workers’ inattention response or behavior can be predicted by assessing biosignals. Kim et al. [73], in their other study, investigated the effectiveness of VR as a behavioral intervention tool to mitigate the decrease in vigilance with habituation to workplace hazards. This safety application target occurred in 26.8% of articles (26 articles). Concerning technologies, 19.6% used VR (19 articles), 2.1% used AR (2 articles), and 7.2% used MR (7 articles).
- **SAO4—Use of heavy equipment and materials.** This type of application trains operators, workers, or students in the correct use or handling of heavy equipment, such as cranes, excavators, forklifts, or materials used in the construction process. There are some examples in the literature. Su et al. [138] performed a VR experience to teach construction excavator control skills. Another research develops a VR-based construction telerobot control system [150]. Barkokebas et al. [143] proposed an approach to evaluate training for the assembly, disassembly, and maintenance of construction machines. Pooladvand et al. [144] developed a crane simulator system in a VR environment. This safety application target appeared in 13.4% of articles (13 articles), all employing VR.

#### 3.2.5. Evaluation within or outside the XR Experience

**Figure 12:**
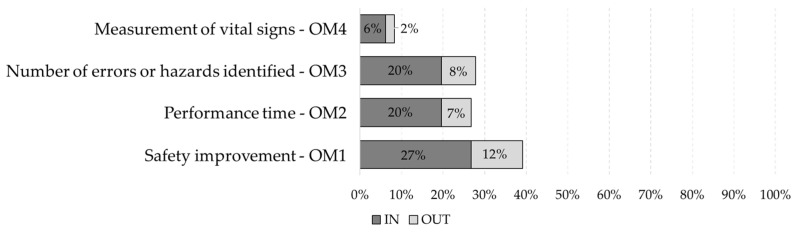
Number of items by type of evaluation of the objective method and number evaluating within and outside the experience.

**Figure 13:**
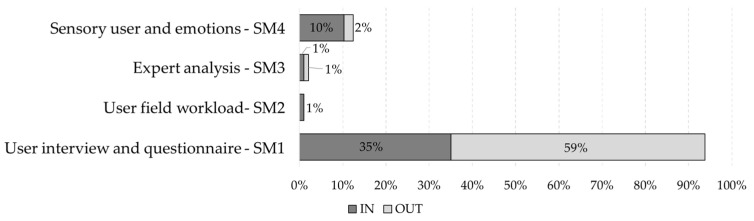
Number of items by type of evaluation of the subjective method and number evaluating within and outside the experience.

This work analyzed and compared the effectiveness evaluation and whether this evaluation occurred outside or within the XR experience developed. This technique revealed the tendencies and how the evaluations are carried out in the various research projects. Figure 12 show the number of items by type of evaluation of the objective method and number evaluating within and outside the experience, and Figure 13 show the number of items by type of evaluation of the subjective method and number evaluating within and outside the experience.

## 4. Discussion

### 4.1. General Discussion

Based on the above results, we seek to determine the primary methods for evaluating the effectiveness of XR experiences, their trends in the literature, and how these methods vary depending on the objective of the safety application of the experience. Based on the results of 129 articles, 32 (24.8%) do not perform an effectiveness evaluation. This result is because they focus on the development of the experience and not on evaluating it. Some of them verify the functioning of the experience but do not mention or provide information on any evaluation, and other cases propose to carry out effectiveness evaluation in future research. There is a higher percentage of evaluation methods for experiences created with VR technology versus AR and MR, which correlates with a similar percentage of publications with research and development on the same technologies. This comes with the chronology of the appearance of the devices required for research.

**Figure 14:**
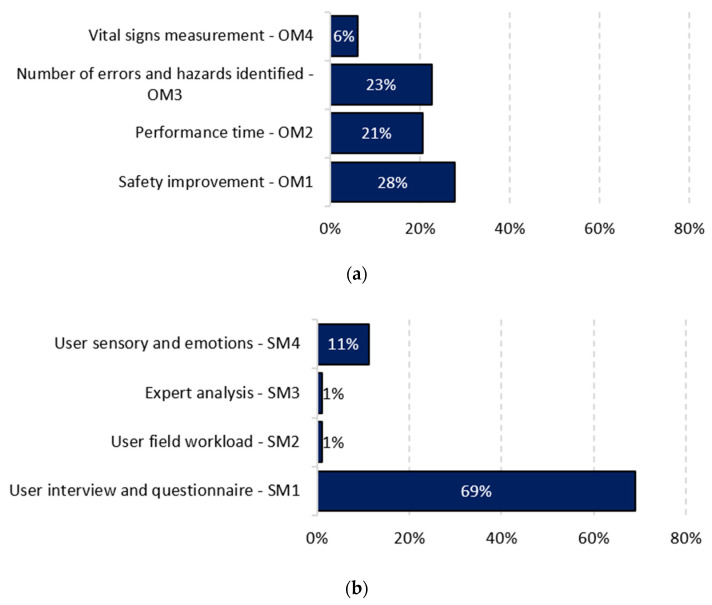
Types of evaluation. (**a**) Objective methods; (**b**) Subjective methods.

Concerning the evaluation methods identified in the literature, there are objective and subjective methods. Figure 14 shows a summary of the results of the four types of objective evaluation and the four types of subjective evaluation identified. Among the objective methods, there are four types of evaluation: ‘Safety improvement’ (28%), ‘Action time’ (21%), ‘Number of errors or hazards identified’ (23%), and ‘Measurement of vital signs’ (6%). For the objective method, the ‘Safety improvement’ type is used most often. This is because it has a higher percentage of articles that use this type of evaluation among the types that represent the objective method. This statistic is consistent, since one of the main objectives of the analyzed items is to contribute in some way to safety within the construction site, for example, by implementing protective equipment or training, which improves workers’ awareness to a certain level and reduces the risks to which they may be exposed, thus improving the overall safety on site. For this reason, comparing the effectiveness of XR experiences to traditional methods validates the use of the experiences.

Likewise, the subjective method uses four types of evaluation: ‘User interview and questionnaire’ (69%), ‘User field workload’ (1%), ‘Expert analysis’ (1%), and ‘Sensory user and emotions’ (11%). The ‘User interview and questionnaire’ evaluation is the most used in the subjective method and the literature in general.

One can evaluate any experience using questions to collect information. It is a fast, low-cost method and can cover many topics within an evaluation because it can gather information from the participants, such as their opinion, knowledge, feedback on usability, applicability, and emotions. Concerning the evaluation type ‘User field workload’, only 1% of articles use this type (equivalent to only one research paper). This means that the literature has not thoroughly investigated the effects of user field workload on construction site safety using XR experiences. The purpose of this evaluation was ‘Monitoring the on-site environment’, and the safety application objective where it was identified was ‘Hazard response and communication’. This result is reasonable, as the cognitive response of participants to hazards within the experience is monitored. ‘Expert analysis’, with 1% of articles using this type of evaluation (which also equates to a single investigation), helps validate the experiences using the opinion or feedback of professionals or industry experts. The fact that this type of evaluation only has one appearance in the literature means that it is a method that is little used or valued by the authors. The reason is probably that no means has been found of obtaining quantifiable value from which to analyze the trends or variables that affect the effectiveness of the experience. As expected, evaluations that employ sensors, such as ‘Vital Signs Measurement’ and ‘Sensory User and Emotions’, are scarce in the literature, as they can be very costly and challenging to implement, which limits research.

The ‘Vital Signs Measurement’ evaluation provides valuable information when identifying the situations that can generate increased stress or fatigue for users to avoid or reduce such situations within the construction site, thus increasing the performance and comfort of workers when performing their work. Concerning XR experiences, this type of evaluation provides body information that can bring experiences closer to reality, in addition to testing which types of sensors or tools of this type are more effective in measuring user responses to various situations.

In terms of safety-related purposes, the literature identified three purposes: ‘Education and training’ (87%), ‘Monitoring the on-site environment’ (8%), and ‘Safety planning’ (5%). Within the literature, the purpose that occurred most often was ‘Education and training’. Therefore, the main focus of research over the years has been to develop XR experiences that assist in the learning and training of workers and students. This process is the first step in improving safety within the construction site, since most accidents arise from the lack of awareness of the participating agents within a project and the misuse of heavy machinery or safety materials, such as personal protective equipment.

In terms of safety application objectives, four types were identified: ‘Hazard avoidance’ (14%), ‘Hazard identification’ (45%), ‘Hazard response and communication’ (27%), and ‘Use of heavy equipment and materials’ (13%). The one that occurred the most within the database was ‘Hazard Identification’. This result is evidence that the authors focus on developing experiences that help improve the perception of risks and hazards within the construction site by creating training or experiences where participants’ reactions to being under these simulated hazards are measured.

Considering whether the evaluation is within or outside the experience, most evaluations occur within. The exception is ‘User interview and questionnaire’, which has more evaluations performed outside the XR experience. It is more common to perform questions, surveys, or questionnaires at the end of the activity to measure what the participant has learned. On the other hand, the other types of evaluation are more effective within the experiences. An example is ‘Performance time’, since it is possible to estimate the time for the activity to be completed or a task or objective to be fulfilled. The same occurs with the ‘Number of errors or hazards identified’, where it is necessary to measure the number while the experience is ongoing. Some cases should use more than one evaluation method. This technique can evaluate the reaction or efficiency of the participants at the time of completing the activity. Later, the knowledge acquired and retained by the users after a predefined time after the end of the experience can be evaluated.

### 4.2. Practical Applications

The use of virtual reality (VR), augmented reality (AR), and mixed reality (MR) technologies not only provides dynamic and eye-catching experiences for consumers, but they are also innovative and technological methods. This novelty makes them attractive to users and challenges them to constantly learn and develop their skills to achieve the objectives proposed for each experience. It is essential to measure the effectiveness of the experiments or situations to define whether the experience is a success or failure or to estimate the main limitations, scopes, or improvements for future research.

Researchers and developers of XR experiences can use the types of evaluation identified and perform a critical analysis of which type of evaluation would be most effective to implement to validate their XR experiences, depending on the purpose and application objectives of their research. To this end, it is essential to refer to some of the articles in the literature analyzed, which makes it easier to complement their research and develop efficient and effective experiences.

### 4.3. Limitations

Although the results and conclusions of this research are based on a systematic literature review and the collection of data related to the objective of this study, there are some limitations of the work performed.

- Although this criterion may eliminate related articles, it is assumed that publications within the last 12 years are sufficient to represent the trends related to the focus of this research, which are extended reality technologies, an emerging technology that has been on the rise within the last decade.
- The methods and types of evaluation identified are based on scientific publications related to the AECO industry, according to the inclusion and exclusion criteria established within the methodology. Therefore, the methods used within other industries may expand or complement the safety-related findings of this research.
- This work does not address the methods to evaluate the performance of hardware tools used within XR experiences and simulations, such as motion sensitivity in virtual reality goggles or sensor accuracy.

## 5. Conclusions

This research gathers evidence of how extended-reality-based experiences in construction have been evaluated over the last 12 years. The use of XR technologies has been increasing over the years, achieving considerable growth in recent years, from 2019 to 2021, so the same trend is expected for 2022 and the following years. The most used technology during the same years is virtual reality. Only some studies use augmented reality or mixed reality. Most of the analyzed documents focus on using these innovative technologies in training their workers, since they provide efficient and interactive experiences when educating and training new and experienced personnel. In evaluating the effectiveness of the experience, out of a total of 97 articles that performed evaluations, two methods and eight types were found. Some papers used more than one type of assessment.

The methods and types of evaluation identified in this research, along with their respective examples, can help future research and highlight the methods used in previous studies. In addition, they give an idea of how to evaluate the effectiveness of different XR experiences that may be developed in future studies, considering the purpose and application objectives as the bases for the experiences. Including methods to evaluate the experiences developed within an investigation is critical. These methods validate the effectiveness of the XR experience and the fulfillment of its objectives. In addition, generating instances of evaluation can identify areas that need improvement to increase the efficiency of the XR experience.

This work recommends using more than one type of evaluation for better effectiveness of XR experiences. For example, in the case of an XR training experience, where the objective is the safety learning of workers, it is proposed to use the ‘Safety improvement’ type of evaluation to compare the increase in safety between traditional training and applying XR. Next, the ‘Number of errors or hazards identified’ quantifies the number of unsafe situations or responses that the participants obtain within the simulation. Finally, the ‘User interview and questionnaire’ observes the knowledge acquired by the participants and personal opinions regarding the functionality and applicability of the XR experience. 

It is recommended for future research to analyze other areas, such as health, commerce, and transportation. It would be helpful to identify the methods used and determine if they complement those already established within this research. This technique would discover innovative methods that can be adapted to other types of experiences to be developed and evaluated. In addition, future work should propose an evaluation standard depending on the purpose, objective, and type of security application, thus making future experiences more effective and easier to validate within the proposed standard.

## Figures and Tables

**Table 1:** Main academic sources identified with two or more articles.

Title of the Journal/Conference	Number of Papers
Automation in Construction	15
Proceedings of the 36th International Symposium on Automation and Robotics in Construction, ISARC 2019	8
Advanced Engineering Informatics	7
Journal of Construction Engineering and Management	5
Safety Science	5
Construction Research Congress 2018: Safety and Disaster Management	4
International Journal of Environmental Research and Public Health	3
Engineering, Construction and Architectural Management	3
International Journal of Occupational Safety and Ergonomics	3
Computing in Civil Engineering 2019: Visualization, Information Modeling, and Simulation	3
Sensors	2
Sustainability	2
Journal of Computing in Civil Engineering	2
Applied Sciences (Basel)	2
Applied Ergonomics	2
Accident Analysis and Prevention	2
Buildings	2
International Journal of Engineering Education	2
WIT Transactions on the Built Environment	2
Journal of Physics: Conference Series	2
Construction Research Congress 2020: Safety, Workforce, and Education—Selected Papers from the Construction Research Congress 2020	2
Construction Research Congress 2020: Computer Applications—Selected Papers from the Construction Research Congress 2020	2

## Data Availability

Not applicable.
